# Upper normal serum magnesium is associated with a reduction in incident death from fatal heart failure, coronary heart disease and stroke in non-dialysis patients with CKD stages 4 and 5

**DOI:** 10.1093/ckj/sfae390

**Published:** 2024-12-02

**Authors:** Cayetana Moyano-Peregrin, Cristian Rodelo-Haad, Alejandro Martín-Malo, Juan Rafael Muñoz-Castañeda, Raquel Ojeda, Isabel Lopez-Lopez, Mariano Rodríguez, Mª Victoria Pendon-Ruiz de Mier, Rafael Santamaría, Sagrario Soriano

**Affiliations:** Maimónides Biomedical Research Institute of Cordoba (IMIBIC-GC13 Calcium Metabolism and Vascular Calcification), Cordoba, Spain; University of Cordoba, Cordoba, Spain; Nephrology Service, Reina Sofia University Hospital, Cordoba, Spain; Spanish Renal Research Network (REDinREN), Institute of Health Carlos III, Madrid, Spain; Maimónides Biomedical Research Institute of Cordoba (IMIBIC-GC13 Calcium Metabolism and Vascular Calcification), Cordoba, Spain; University of Cordoba, Cordoba, Spain; Nephrology Service, Reina Sofia University Hospital, Cordoba, Spain; Spanish Renal Research Network (REDinREN), Institute of Health Carlos III, Madrid, Spain; Maimónides Biomedical Research Institute of Cordoba (IMIBIC-GC13 Calcium Metabolism and Vascular Calcification), Cordoba, Spain; University of Cordoba, Cordoba, Spain; Nephrology Service, Reina Sofia University Hospital, Cordoba, Spain; Spanish Renal Research Network (REDinREN), Institute of Health Carlos III, Madrid, Spain; Maimónides Biomedical Research Institute of Cordoba (IMIBIC-GC13 Calcium Metabolism and Vascular Calcification), Cordoba, Spain; University of Cordoba, Cordoba, Spain; Nephrology Service, Reina Sofia University Hospital, Cordoba, Spain; Spanish Renal Research Network (REDinREN), Institute of Health Carlos III, Madrid, Spain; Maimónides Biomedical Research Institute of Cordoba (IMIBIC-GC13 Calcium Metabolism and Vascular Calcification), Cordoba, Spain; University of Cordoba, Cordoba, Spain; Nephrology Service, Reina Sofia University Hospital, Cordoba, Spain; Spanish Renal Research Network (REDinREN), Institute of Health Carlos III, Madrid, Spain; Maimónides Biomedical Research Institute of Cordoba (IMIBIC-GC13 Calcium Metabolism and Vascular Calcification), Cordoba, Spain; University of Cordoba, Cordoba, Spain; Nephrology Service, Reina Sofia University Hospital, Cordoba, Spain; Spanish Renal Research Network (REDinREN), Institute of Health Carlos III, Madrid, Spain; Maimónides Biomedical Research Institute of Cordoba (IMIBIC-GC13 Calcium Metabolism and Vascular Calcification), Cordoba, Spain; University of Cordoba, Cordoba, Spain; Nephrology Service, Reina Sofia University Hospital, Cordoba, Spain; Spanish Renal Research Network (REDinREN), Institute of Health Carlos III, Madrid, Spain; Maimónides Biomedical Research Institute of Cordoba (IMIBIC-GC13 Calcium Metabolism and Vascular Calcification), Cordoba, Spain; University of Cordoba, Cordoba, Spain; Nephrology Service, Reina Sofia University Hospital, Cordoba, Spain; Spanish Renal Research Network (REDinREN), Institute of Health Carlos III, Madrid, Spain; Maimónides Biomedical Research Institute of Cordoba (IMIBIC-GC13 Calcium Metabolism and Vascular Calcification), Cordoba, Spain; University of Cordoba, Cordoba, Spain; Nephrology Service, Reina Sofia University Hospital, Cordoba, Spain; Spanish Renal Research Network (REDinREN), Institute of Health Carlos III, Madrid, Spain; Maimónides Biomedical Research Institute of Cordoba (IMIBIC-GC13 Calcium Metabolism and Vascular Calcification), Cordoba, Spain; University of Cordoba, Cordoba, Spain; Nephrology Service, Reina Sofia University Hospital, Cordoba, Spain; Spanish Renal Research Network (REDinREN), Institute of Health Carlos III, Madrid, Spain

**Keywords:** chronic kidney disease, coronary heart disease, heart failure, magnesium, nutrition

## Abstract

**Background:**

Serum magnesium disturbances are common in patients with cardiovascular disease (CVD). However, the well-established link between low serum magnesium and nutritional or inflammatory disorders has limited its consideration as a non-traditional risk factor for mortality. This study aims to elucidate the relationship between serum magnesium concentrations and mortality due to fatal heart failure (HF), coronary heart disease (CHD) and stroke in non-dialysis patients with chronic kidney disease (CKD) stages 4 and 5.

**Methods:**

A cohort of 1271 non-dialysis patients with CKD stages 4 and 5 was followed from 2008 to 2018. Patients with prior major adverse cardiovascular events (MACE) were excluded. Serum magnesium levels were stratified into tertiles and the primary outcomes were incidence rates of fatal HF, CHD and stroke. Secondary outcomes included composite MACE and all-cause mortality. Hazard ratios (HRs) were calculated using multivariate Cox regression, adjusting for demographics, comorbidities and biochemical parameters. E-values were used to assess the robustness of the results.

**Results:**

Over the 10-year follow-up, 186 patients died. Higher serum magnesium levels were significantly associated with reduced mortality risk from HF [HR 0.49 (95% CI 0.27–0.89) for T2; HR 0.31 (95% CI 0.16–0.60) for T3] compared with the lowest tertile. Similar trends were observed for CHD and stroke mortality. The incidence rate of MACE per 1000 person-years was reduced from 68.2 in tertile 1 to 26.2 in tertile 2 and 16.8 in tertile 3. Secondary endpoints, including all-cause mortality and composite MACE, followed trends similar to the primary outcomes.

**Conclusions:**

Higher serum magnesium concentrations were associated with lower risks of death from fatal HF, CHD and stroke in non-dialysis patients with CKD stages 4 and 5.

## Graphical Abstract

**Graphical Abstract:**
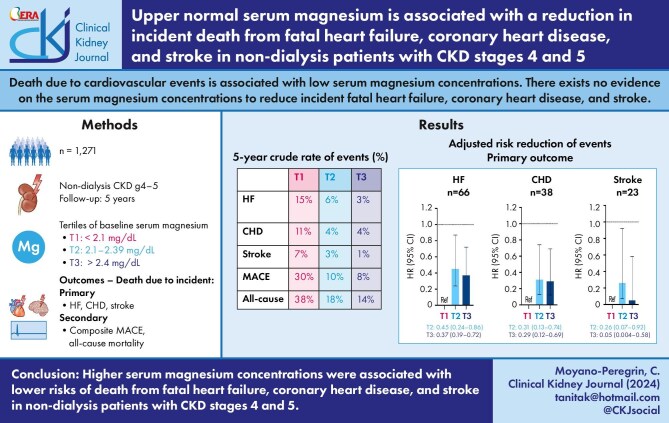

KEY LEARNING POINTS **What was known:**

- Low serum magnesium levels are associated with cardiovascular disease.
- However, limited data exist regarding the serum magnesium level associated with reduced cardiovascular mortality in patients with non-dialysis chronic kidney disease (CKD).

**This study adds:**

- In this observational study involving 1271 non-dialysis CKD patients, baseline serum magnesium concentrations >2.1 mg/dl were associated with a lower incidence of death from fatal heart failure (HF), coronary heart disease (CHD) and stroke.
- The association of lower serum magnesium levels with major adverse cardiovascular events remains significant despite the effects of concomitant low albumin and high CRP as surrogates of poor nutrition and inflammation, which may contribute to a reduction in serum magnesium concentration.

**Potential impact:**

- Given the association between low serum magnesium concentrations and an increased risk of HF, CHD and stroke, optimal care should aim to prevent or correct decreases in serum magnesium among patients with CKD.

## INTRODUCTION

Cardiovascular disease (CVD) represents the primary cause of mortality among patients with chronic kidney disease (CKD) [1]. Despite the implementation of strategies targeting established risk factors for CVD, such as hypertension, dyslipidaemia, smoking and diabetes, the observed improvement in survival remains inadequate. Emerging factors such as vascular calcifications, inflammation and proteinuria also contribute to the persistent mortality risk [2]. Serum magnesium has been identified as a predictor of cardiovascular disease (CVD) due to its role in regulating enzymatic and cellular functions [3–6]. A correlation has been identified between serum magnesium levels and mortality, with low levels being associated with a higher mortality risk among healthy individuals [7]. Magnesium deficiency is uncommon in CKD because of impaired renal magnesium excretion [8]. Indeed, in patients with advanced CKD, reports indicate that only 14% of patients exhibit low or low-normal magnesium concentrations [9]. The use of proton pump inhibitors (PPIs) and diuretics, comorbidities such as diabetes, volume overload, overt inflammation and nutritional imbalances may account for the majority of cases of magnesium deficiency observed in this population [4]. Mizuiri *et al.* [10] demonstrated that serum magnesium levels <2.4 mg/dl were linked to an increased risk of mortality from both non-cardiovascular and cardiovascular causes in patients undergoing dialysis. Furthermore, another study indicated that the association between hypomagnesemia and cardiovascular mortality in dialysis patients was influenced by the presence of inflammation [11]. In patients with diabetes and normal renal function, maintaining serum magnesium concentrations within the upper-normal range was associated with a reduced risk of fatal heart failure (HF) and CKD [12]. Both experimental [13] and clinical evidence [14] support the role of magnesium in the mitigation of vascular calcification. However, there is limited scientific literature identifying the serum magnesium concentration associated with reduced cardiovascular mortality in patients with advanced non-dialysis CKD.

The present study aims to elucidate the association between serum magnesium concentration and the incidence of death from fatal HF, coronary heart disease (CHD) and stroke. Additionally, we assess the incidence of composite major adverse cardiovascular events (MACE) and all-cause mortality in patients with CKD stages 4 and 5. Finally, we analyse the extent to which coexisting nutritional and inflammatory disorders influence the relationship between magnesium levels and mortality.

## MATERIALS AND METHODS

### Study population

This observational study included 1655 consecutive patients who were regularly followed up at our advanced CKD outpatient clinic between 1 January 2008 and 31 December 2018. A total of 1271 patients met the following inclusion criteria: they were required to have undergone a minimum of 3 months of follow-up to ensure that their estimated glomerular filtration rate (eGFR) remained <30 ml/min/1.73 m^2^ for the entire observation period. Furthermore, they were required to show no clinical signs of malignancy or other debilitating disease (Supplementary Fig. S1). Patients who had previously experienced a non-fatal MACE were excluded from the study (*n* = 182). Participants were followed until one of the following events occurred: death, initiation of renal replacement therapy, loss to follow-up, end of follow-up or until the database was locked for analysis on 31 December 2018. The data were collected by the Nephrology Department’s electronic database (Reina Sofia University Hospital). This research protocol was conducted in accordance with the 1975 Declaration of Helsinki, as revised in 2013, and received approval from the local ethics committee (Comité de Ética de la Investigación de Córdoba; ref 4962; protocol FIBREMAG).

### Outcomes

The primary endpoint was the analysis of mortality resulting from incident HF, CHD or stroke. The diagnosis of HF was made following predefined criteria [15, 16] and was confirmed by specific medical record entries. CHD was defined as death resulting from the occurrence of an acute myocardial infarction, as previously described [17]. This definition encompassed clinical symptoms indicative of myocardial ischaemia, alterations in the electrocardiogram and the mobilisation of cardiac troponin. A stroke was defined by both clinical and imaging evidence of brain ischaemia [18]. The endpoints of death resulting from CHD or stroke were obtained from medical records. The secondary endpoints were the composite of deaths from each subtype of MACE and all-cause mortality accounting for baseline serum magnesium concentrations, which were categorised into tertiles. All-cause mortality was defined as death from any cause.

### Covariates

Demographic, treatment and biochemical data, including serum magnesium, were collected at baseline. Diabetes was defined according to previously established criteria [19]. The eGFR was calculated using the Chronic Kidney Disease Epidemiology Collaboration (CKD-EPI) equation [20]. The first-morning void urine sample was used to calculate the urinary protein:creatinine ratio (UPCR). High-sensitivity C-reactive protein (CRP) levels were quantified by an immunoturbidimetric CRP-Latex (II) assay, with a reference range of 0–10 mg/l. Serum parathyroid hormone (PTH) levels were assessed by immunoradiometric assay (coated bead; Scantibodies Laboratory, Santee, CA, USA), with a laboratory reference range of 15–65 pg/ml. Serum albumin levels were measured by immunoturbidimetry (bromocresol purple) with a laboratory reference range of 3.2–4.8 g/dl. Standard biochemical methods were employed to determine magnesium (reference range 1.7–2.7 mg/dl) and other analytes.

### Statistical analysis

Continuous variables are presented as means, standard deviations (SDs), medians and interquartile ranges (IQRs, 25th–75th percentiles). Categorical variables are expressed as percentages. Spearman's correlation test was used to assess associations between numerical variables. A chi-squared or Fisher's exact test was used to compare patient demographics, clinical parameters and laboratory values between groups for categorical variables, while a *t*-test or Wilcoxon rank-sum test was used for continuous variables.

Given the potential non-linear relationship between serum magnesium concentration and mortality, magnesium levels were categorised into tertiles. In additional analyses, the serum magnesium concentration was used as a continuous variable using a restricted cubic spline function with three knots to model non-linear effects. Based on serum magnesium tertiles, the Kaplan–Meier estimate of death probability was compared using the logrank test. Univariate and multivariate Cox proportional hazards analyses assessed predictors of mortality for each outcome. Missing covariate values were imputed using the multiple imputation by chained equations (MICE) algorithm. A total of five imputations were performed. The results of these imputations were combined to produce robust estimates [21].

Models with baseline serum magnesium were categorised into tertiles. Model 1 was adjusted for sex, age, diabetes, hypertension, body mass index (BMI), tobacco use and tertiles of magnesium. Model 2 was adjusted for significant variables identified in model 1 (tertiles of magnesium and BMI), serum phosphate, haemoglobin, the use of oral iron preparations, calcium binders, renin–angiotensin–aldosterone system (RAAS) acting agents, statins, aspirin and beta-blockers. Model 3 was adjusted for significant variables in model 2 (magnesium tertiles, BMI), intact PTH (iPTH), serum calcium, high-sensitivity CRP (hs-CRP), diuretics, vitamin K antagonist (VKA) and calcium channel blockers. Model 4 was adjusted for significant variables in model 3 (magnesium tertiles, iPTH and diuretics), eGFR, serum albumin, hs-CRP, albumin*hs-CRP interaction, total cholesterol and the use of PPIs. Multivariable models with serum magnesium concentration as a continuous variable included the same variables plus interaction terms in different models (see Supplementary Methods). CRP and serum albumin levels were initially included in separate models to avoid collinearity. Additional models included CRP and serum albumin together to assess their interaction. Treatment variables were included as binary indicators (yes/no). The protein-energy wasting syndrome (PEW) expert panel recommends serum albumin >3.8 g/dl and CRP 10 mg/l as cut-off values to support nutrition and inflammation in CKD [22, 23]. Therefore, we constructed four real-life clinical nutrition and inflammation profiles as follows: normal albumin and normal CRP (albumin >3.8 g/dl, CRP <10 mg/l), normal albumin and high CRP (albumin >3.8 g/dl, CRP >10 mg/l), low albumin and normal CRP (albumin <3.8 g/dl, CRP <10 mg/l) and low albumin and high CRP (albumin <3.8 g/dl, CRP >10 mg/l). The least squares mean method, adjusted for age, eGFR and serum phosphate as a surrogate for protein intake, was employed to analyse the effect of these variables on serum magnesium concentration. This was achieved by comparing the concentrations across different nutritional/inflammatory clinical profiles. A further multivariable Cox model was constructed, incorporating nutritional and inflammatory profiles as follows: model 5 was adjusted for significant variables in model 4 (magnesium tertiles, iPTH, total cholesterol and diuretics), UPCR, the use of PPIs, the interaction between PPIs and diuretics and nutritional/inflammatory profiles (normal albumin/normal CRP as the reference category). To ensure the robustness of the results, we performed prespecified subgroup analyses in patients with echocardiographic imaging data and adjusted for the presence of left ventricular hypertrophy (LVH), left ventricular ejection fraction (LVEF), mitral annular calcification (MAC) and aortic valve calcification (AVC). The confounding variables included in each model are described in the Supplementary Methods. E-value calculations were used to determine the minimum strength of unmeasured confounding factors required to negate the effect of magnesium on mortality [24, 25]. *P*‐values <.05 were considered statistically significant. Statistical analyses were performed using R version 4.2.2 (R Foundation for Statistical Computing, Vienna, Austria) [26].

## RESULTS

### Characteristics of the population

The study included 1271 patients with CKD due to hypertension [26% (*n* = 331)], diabetes [20% (*n* = 260)], polycystic kidney disease [8.9% (*n* = 113)] and primary glomerulonephritis [8.1% (*n* = 103)]. Additionally, 28% (*n* = 353) of patients had CKD from unknown causes, while 8.7% (*n* = 111) had CKD due to specific conditions such as amyloidosis, multiple myeloma and urinary tract infections.

The median follow-up period was 2.1 years (IQR 0.79–3.44). During this time, 186 patients (14.6%) died, with 127 of these deaths (68.3%) attributed to MACE. The remaining 31.7% (*n* = 59) died from other causes, including 29 cases of abdominal/urinary-associated septic shock, 23 cases of pneumonia, 3 cases of neoplasia, 3 cases of upper gastrointestinal bleeding and 1 case of convulsive status epilepticus.

The mean baseline serum magnesium concentration was 2.21 mg/dl (range 1.0–4.70 mg/dl). Table 1 shows baseline characteristics and laboratory values according to tertiles of magnesium concentration. Comparisons between tertiles showed significant differences in age, eGFR, serum albumin levels, systolic blood pressure (SBP), diastolic blood pressure (DBP) and UPCR.

**Table 1:** Characteristics of the population according to baseline magnesium tertiles.

			Magnesium tertiles				
Characteristics	All (*N* = 1271)	Missing data	T1 <2.1 mg/dl (*n* = 410)	T2 2.1–2.39 mg/dl (*n* = 430)	T3 >2.4 mg/dl (*n* = 431)	*P*-value	SMD
Sex, *n* (%)							
Female	488 (38.4)	0	131 (32.0)	161 (37.4)	196 (45.5)	<.001	0.187
Male	783 (61.6)	0	279 (68.0)	269 (62.6)	235 (54.5)		
DM, *n* (%)							
No	836 (65.8)	0	249 (60.7)	299 (69.5)	288 (66.8)	.023	0.124
Yes	435 (34.2)	0	161 (39.3)	131 (30.5)	143 (33.2)		
Hypertension, *n* (%)							
No	413 (32.5)	0	127 (31.0)	141 (32.8)	145 (33.6)	.705	0.038
Yes	858 (67.5)	0	283 (69.0)	289 (67.2)	286 (66.4)		
Tobacco, *n* (%)							
No	558 (65.3)	392	182 (62.8)	190 (64.6)	186 (63.1)	.880	0.026
Yes	321 (36.5)		108 (37.2)	104 (35.4)	109 (36.9)		
Follow-up (years), median (IQR)	2.14 (0.79–4.33)	0	1.94 (0.69–3.53)	2.22 (0.88–4.54)	2.37 (0.88–5.23)	.001	0.202
Age (years), mean (SD)	72.10 (13.31)	0	70.93 (12.56)	70.82 (13.75)	74.50 (13.27)	<.001	0.186
eGFR by CKD-EPI (ml/min/1.73 m^2^), mean (SD)	15.03 (4.78)	0	15.64 (4.83)	15.22 (4.72)	14.25 (4.71)	<.001	0.195
BMI (kg/m), mean (SD)	29.15 (4.48)	0	29.31 (4.19)	29.03 (4.61)	29.11 (4.61)	.638	0.043
SBP (mmHg), mean (SD)	147.15 (22.04)	196	149.31 (21.60)	145.05 (22.24)	147.18 (22.12)	.020	0.129
DBP (mmHg), mean (SD)	79.65 (13.00)	196	81.13 (12.32)	79.55 (13.72)	78.34 (12.78)	.008	0.145
MBP (mmHg), mean (SD)	102.15 (13.70)	196	103.86 (13.04)	101.38 (14.41)	101.29 (13.45)	.009	0.127
PP (mmHg), mean (SD)	67.70 (19.45)	196	68.35 (19.40)	65.82 (18.75)	68.97 (20.08)	.042	0.109
Haemoglobin (g/l), mean (SD)	11.54 (1.59)	0	11.48 (1.61)	11.56 (1.60)	11.59 (1.56)	.609	0.045
Haematocrit (%), mean (SD)	34.79 (4.64)	0	34.55 (4.77)	34.81 (4.64)	35.00 (4.52)	.371	0.065
Calcium (mg/dl), mean (SD)	9.19 (0.72)	0	9.03 (0.81)	9.23 (0.63)	9.29 (0.70)	<.001	0.233
Serum phosphate (mg/dl), mean (SD)	4.43 (0.80)	0	4.32 (0.75)	4.39 (0.78)	4.56 (0.86)	<.001	0.203
Serum magnesium (mg/dl), mean (SD)	2.21 (0.42)	0	1.78 (0.22)	2.20 (0.08)	2.64 (0.33)	<.001	2.498
iPTH (pg/ml), median (IQR)	150.0 (90.2–241.0)	66	164.45 (97.5–277.3)	142.25 (84.0–224.7)	145.70 (85.5–231.1)	.005	0.140
Total cholesterol (mg/dl), mean (SD)	176.42 (41.99)	32	173.16 (40.85)	178.70 (42.21)	177.25 (42.74)	.142	0.088
hs-CRP (mg/l), median (IQR)	4.40 (1.80–10.25)	0	4.45 (1.70–10.65)	4.30 (1.8–10.57)	4.30 (1.75–9.70)	.833	0.041
Albumin (g/l), mean (SD)	3.90 (0.51)	0	3.80 (0.53)	3.92 (0.47)	3.98 (0.51)	<.001	0.235
UPCR (mg/g), mean (SD)	1.760 (2.61)	0	2.210 (2.95)	1.560 (2.44)	1.520 (2.35)	<.001	0.173
RAASi, *n* (%)							
No	543 (42.7)	0	174 (42.4)	184 (42.8)	185 (42.9)	.989	0.007
Yes	728 (57.3)		236 (57.6)	246 (57.2)	246 (57.1)		
Diuretics, *n* (%)							
No	622 (48.9	0	215 (52.4)	217 (50.5)	190 (44.1)	.039	0.112
Yes	649 (51.1)		195 (47.6)	213 (49.5)	241 (55.9)		
Calcium-based phosphate binder, *n* (%)							
No	1117 (87.9)	0	361 (88.0)	385 (89.5)	371 (86.1)	.297	0.071
Yes	154 (12.1)		49 (12.0)	45 (10.5)	60 (13.9)		
PPI, *n* (%)							
No	809 (63.7)	0	257 (62.7)	284 (66.0)	268 (62.2)	.442	0.054
Yes	462 (36.3)		153 (37.3)	146 (34.0)	163 (37.8)		
Iron supplements, *n* (%)							
No	904 (71.1)	0	301 (73.4)	318 (74.0)	285 (66.1)	.019	0.114
Yes	367 (28.9)		109 (26.6)	112 (26.0)	146 (33.9)		
Statins, *n* (%)							
No	904 (71.1)	255	137 (41.5)	132 (38.6)	137 (39.8)	.741	0.040
Yes	367 (28.9)		193 (58.5)	210 (61.4)	207 (60.2)		
Aspirin, *n* (%)							
No	609	390	199 (68.6)	210 (70.9)	200 (67.8)	.691	0.046
Yes	272		91 (31.4)	86 (29.1)	95 (32.2)		
ADP antagonist, *n* (%)							
No	892	392	264 (91.0)	268 (90.6)	260 (88.4)	.501	0.057
Yes	87		26 (9.0)	27 (9.2)	34 (11.6)		
VKA, *n* (%)							
No	813	393	267 (92.4)	274 (92.9)	268 (91.2)	.726	0.042
Yes	65		22 (7.6)	21 (7.1)	26 (8.8)		
Calcium channel blockers, *n* (%)							
No	700	391	235 (81)	231 (78)	234 (79.6)	.668	0.050
Yes	180		55 (19)	65 (22)	60 (20.4)		
Beta-blockers, *n* (%)							
No	609	391	192 (66.2)	211 (71.5)	206 (69.8)	.364	0.077
Yes	271		98 (33.8)	84 (28.5)	89 (30.2)		

D.M.: diabetes mellitus; eGFR: estimated glomerular filtration rate by the CKD-EPI formula; BMI: body mass index, calculated as weight in kilograms divided by height in meters squared; MBP: mean arterial blood pressure, calculated as DBP + 1/3(SBP − DBP); P.P.: pulse pressure, calculated as the difference between the SBP and DBP; RAASi: renin–angiotensin–aldosterone system inhibitor.

Patients in the lowest tertile (T1) were more likely to have hypertension and diabetes compared with other tertiles, but the prevalence of tobacco use was similar across groups. Serum magnesium correlated with eGFR (*r* = −0.14, *P* < .001), serum albumin (*r* = 0.15, *P* < .001), age (*r* = 0.13, *P* < .001), iPTH (*r* = 0.09, *P* = .01) and serum phosphate (*r* = 0.14, *P* < .001). However, there was no significant correlation between serum magnesium concentration and CRP levels (*r* = −0.03, *P* = .25) (Supplementary Fig. S2).

### Association of serum magnesium concentration with HF, CHD and stroke

**Figure 1:**
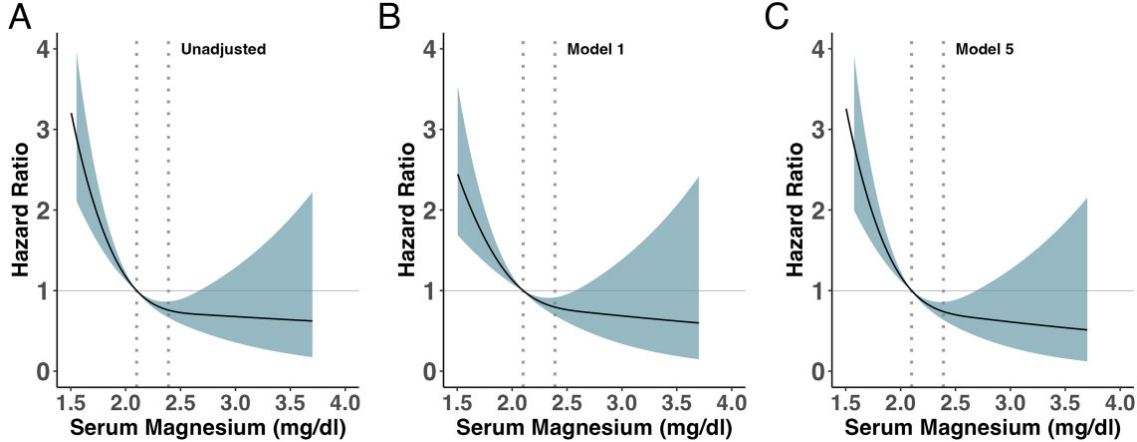
Restricted cubic splines of baseline serum magnesium and their association with MACE. We exclusively show the more relevant models: **(A)** unadjusted model, **(B)** model 1 and **(C)** model 5. The dotted lines represent percentile 33 and percentile 66. The shaded area represents the 95% CI. Model 1 was adjusted for sex, age, diabetes, hypertension, BMI and tobacco use. Model 5 was adjusted for significant variables in model 4 (magnesium tertiles, iPTH, total cholesterol and diuretics) plus UPCR, the use of PPIs, PPIs*diuretics interaction and nutritional/inflammatory profiles (albumin normal/CRP normal as reference).

We used univariable and multivariable restricted cubic splines with three knots to assess the association between serum magnesium concentration and the different outcomes. Deaths due to HF (*n* = 66) were twice as frequent as deaths due to CHD (*n* = 38) and three times more frequent than deaths from stroke (*n* = 23). Higher serum magnesium was significantly associated with a lower risk of fatal HF, CHD and stroke (Supplementary Table S1). MACE-related deaths were reduced in patients with serum magnesium >2.1 mg/dl (Fig. 1A–C). There were no significant interactions between serum albumin and CRP (*P* for interaction = .195), serum magnesium and serum albumin (*P* for interaction = .883), magnesium, CRP and albumin (*P* for interaction = .314) or PPIs and diuretics (*P* for interaction = .837) regarding the association between serum magnesium and the primary and secondary outcomes (Supplementary Table S1). The effect of serum magnesium on all-cause mortality remained significant after adjustment for confounders (Supplementary Table S1). For all-cause mortality, survivors and deceased patients had comparable serum phosphate, albumin, eGFR and UPCR (Supplementary Table S2). Patients who died were more likely to have received calcium channel blockers. The use of statins, adenosine diphosphate (ADP) antagonists, VKA and beta-blockers was not associated with mortality (Supplementary Table S2).

### Risk of fatal HF, CHD and stroke according to serum magnesium tertiles

**Figure 2:**
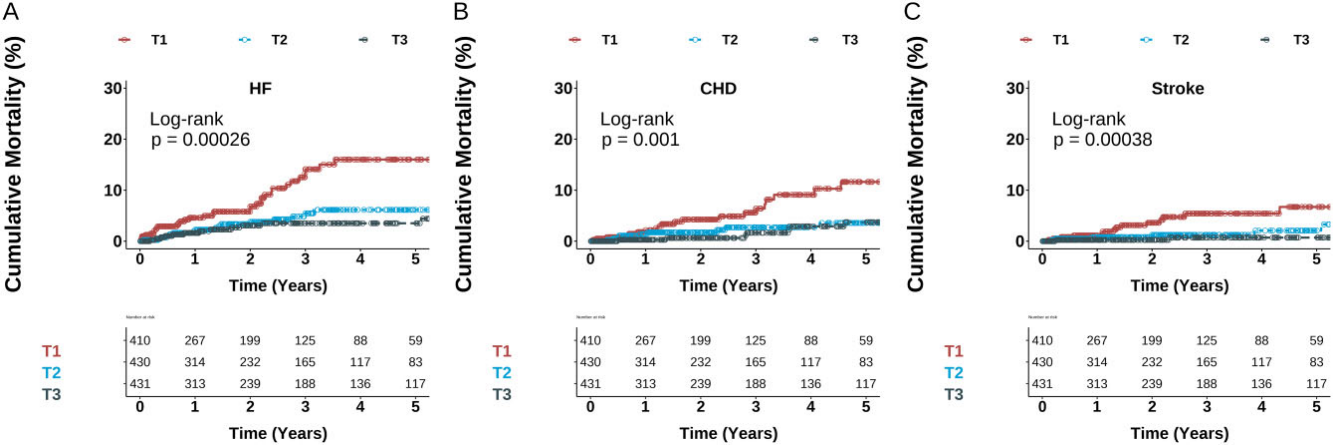
Kaplan–Meier curves for the risk of death due to **(A)** HF, **(B)** CHD and **(C)** stroke according to baseline magnesium tertiles. T1: <2.1 mg/dl, T2: 2.1–2.39 mg/dl, T3: >2.4 mg/dl.

**Figure 3:**
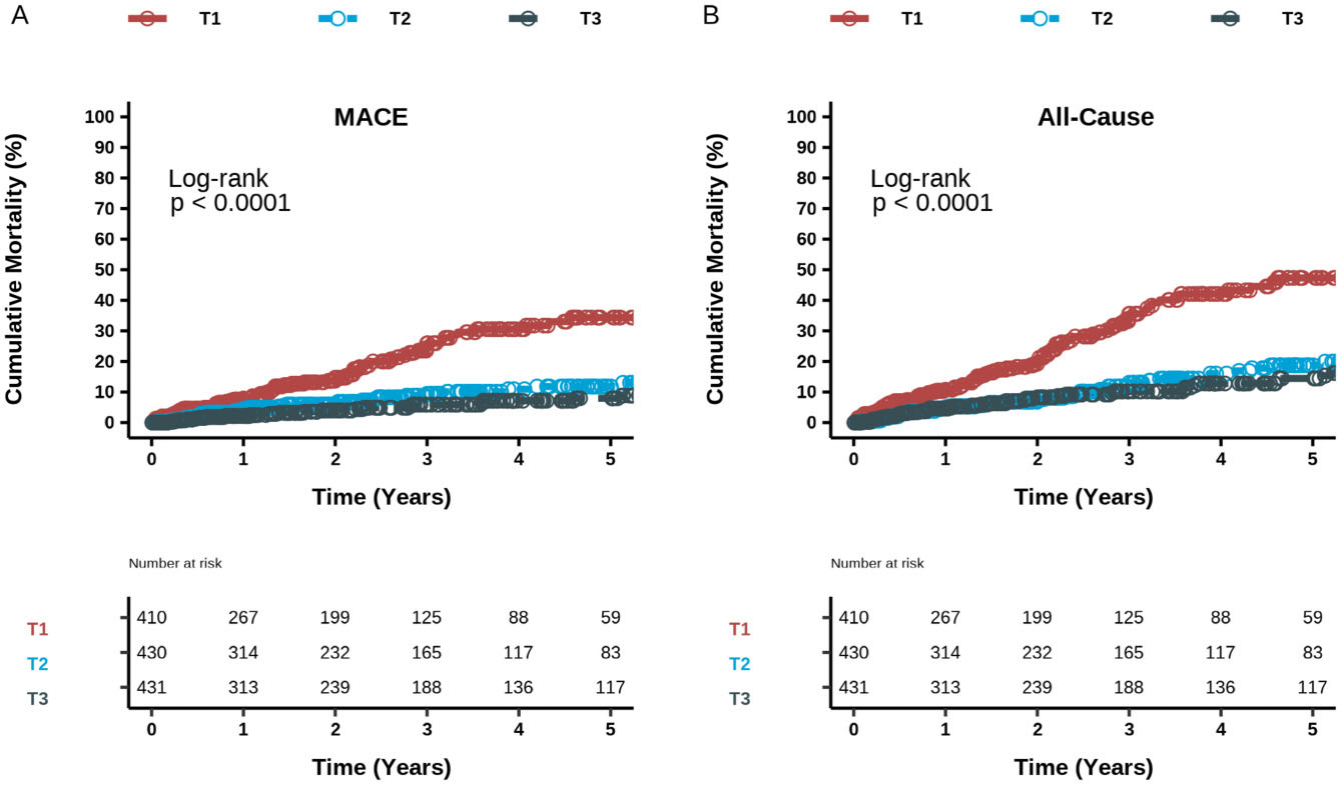
Cumulative death from **(A)** MACE and **(B)** all-cause mortality according to tertiles of baseline serum magnesium. T1: <2.1 mg/dl, T2: 2.1–2.39 mg/dl; T3: >2.4 mg/dl.

**Figure 4:**
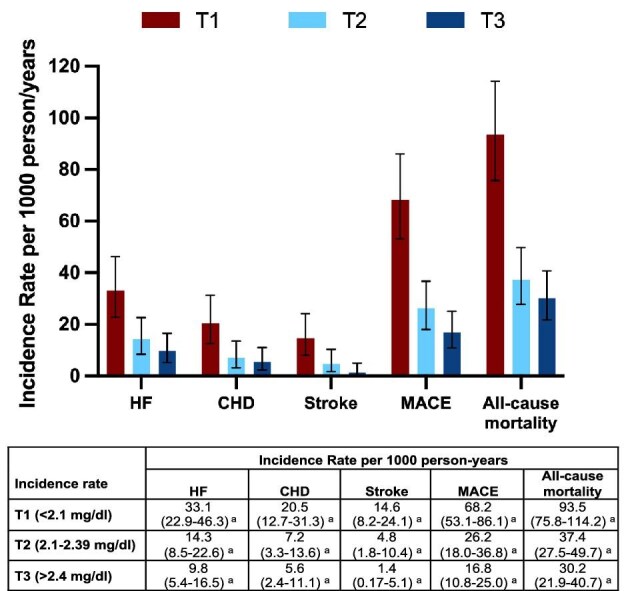
Incidence rate per 1000 person-years according to different outcomes and baseline magnesium tertiles. The incidence rate for subjects within the T2 and T3 in T1 subjects was lower than that in the lowest tertile, irrespective of the outcome. ^a^Data in parentheses are displayed as 95% CIs.

Given the well-established non-linear relationship between serum magnesium concentration and reductions in primary and secondary outcomes, we further analysed the association of serum magnesium concentration with mortality according to serum magnesium tertiles. The 5-year risk of fatal HF decreased from 15% in T1 patients to 6% in T2 and 3% in T3 patients (Fig. 2A). Deaths from CHD and stroke in T1, T2 and T3 showed the same trend as HF, after adjusting for covariates (Fig. 2B and C and Table 2). For the composite MACE, crude mortality declined from 30% in T1 patients to 10% in T2 and 8% in T3 patients (Fig. 3A). Multivariable models confirmed the decreasing risk of MACE in the upper tertiles of serum magnesium (Table 2). Regarding all-cause mortality, patients in the lowest tertile had a 38% incidence of death, which decreased to 13% in T3 patients at year 5 (Fig. 3B). This association persisted across all multivariable models (Table 2). As a result, the crude incidence rate of death per 1000 person-years decreased consistently across tertiles of magnesium (Fig. 4).

**Table 2:** Tertiles of baseline serum magnesium and the risk of clinical outcomes.

Cause of death	Unadjusted	Model 1	Model 2	Model 3	Model 4	Model 5, nutritional/inflammatory profiles
Heart failure (66 events)						
T1	Reference	Reference	Reference	Reference	Reference	Reference
T2	0.45 (0.25–0.80)**	0.50 (0.27–0.91)*	0.54 (0.30–0.99)*	0.52 (0.27–0.98)*	0.51 (0.26–1.0)*	0.45 (0.24–0.86)*
T3	0.32 (0.17–0.61)***	0.35 (0.18–0.67)**	0.36 (0.18–0.69)**	0.40 (0.21–0.79)**	0.38 (0.18–0.80)*	0.37 (0.19–0.72)**
Coronary heart disease (38 events)						
T1	Reference	Reference	Reference	Reference	Reference	Reference
T2	0.35 (0.16–0.79)*	0.43 (0.19–1.0)*	0.41 (0.18–0.97)*	0.36 (0.15–0.87)*	0.31 (0.12–0.74)*	0.31 (0.13–0.74)*
T3	0.28 (0.12–0.66)**	0.33 (0.14–0.79)*	0.33 (0.14–0.79)*	0.31 (0.13–0.77)**	0.28 (0.11–0.69)**	0.29 (0.12–0.69)**
Stroke (23 events)						
T1	Reference	Reference	Reference	Reference	Reference	Reference
T2	0.33 (0.12–0.92)*	0.38 (0.13–1.12)	0.38 (0.12–1.22)	0.32 (0.09–1.09)	0.33 (0.08–1.28)	0.26 (0.07–0.92)*
T3	0.10 (0.02–0.50)**	0.12 (0.02–0.63)*	0.12 (0.02–0.70)*	0.06 (0.05–0.53)*	0.06 (0.004–0.87)*	0.05 (0.004–0.58)*
MACE (127 events)						
T1	Reference	Reference	Reference	Reference	Reference	Reference
T2	0.39 (0.26–0.60)***	0.45 (0.30–0.70)***	0.48 (0.31–0.73)***	0.43 (0.27–0.68)***	0.41 (0.25–0.65)***	0.38 (0.24–0.60)***
T3	0.26 (0.16–0.42)***	0.30 (0.18–0.48)***	0.31 (0.19–0.50)***	0.30 (0.18–0.49)***	0.28 (0.16–0.48)***	0.28 (0.17–0.45)***
All-cause mortality (186 events)						
T1	Reference	Reference	Reference	Reference	Reference	Reference
T2	0.41 (0.29–0.58)***	0.47 (0.33–0.61)***	0.48 0.34–0.69)***	0.47 0.32–0.68)***	0.46 0.31–0.67)***	0.43 0.29–0.63)***
T3	0.34 (0.24–0.49)***	0.39 (0.27–0.56)***	0.39 (0.27–0.57)***	0.41 (0.28–0.60)***	0.39 (0.26–0.58)***	0.37 (0.25–0.54)***

Model 1 adjusted for sex, age, diabetes, hypertension, BMI and tobacco use. Model 2 adjusted for significant variables in model 1 (magnesium tertiles and BMI) plus serum phosphate, haemoglobin and the use of oral iron preparations, calcium binders, RAAS inhibitors, statins, aspirin and beta-blockers. Model 3 adjusted for significant variables in model 2 (magnesium tertiles, BMI) plus iPTH, serum calcium, hs-CRP, diuretics, VKAs and calcium channel blockers. Model 4 adjusted for significant variables in model 3 (magnesium tertiles, iPTH and diuretics) plus eGFR, serum albumin, hs-CRP, albumin*hs-CRP interaction, total cholesterol and the use of PPPIs. Model 5 adjusted for significant variables in model 4 (magnesium tertiles, iPTH, total cholesterol and diuretics) plus UPCR, the use of PPIs, PPI*diuretics interaction and nutritional/inflammatory profiles (albumin normal/CRP normal as reference). *P < .05, **P < .01, ***P < .001.

The results are presented as hazard ratios (95% confidence intervals) compared with the reference group. T1: <2.1 mg/dl, T2: 2.1–2.39 mg/dl, T3: >2.4 mg/dl.

### Effect of albumin and CRP on the relationship between serum magnesium levels and fatal HF, CHD and stroke

We generated nutritional/inflammatory profiles based on serum concentrations of albumin and CRP. These profiles were used to test the potential effect of nutritional status and inflammation on the association of serum magnesium concentrations with the risk of specific outcomes. Supplementary Table S3 describes the characteristics of the four nutritional/inflammatory profiles. A significant but subtle difference in adjusted serum magnesium concentrations was observed between the normal albumin–normal CRP profile and the low albumin–high CRP profile (2.24 ± 0.39 mg/dl versus 2.14 ± 0.47 mg/dl; *P* = .003) (Supplementary Fig. S3). There were 89 deaths out of 666 patients (13.3%) in the normal albumin–normal CRP profile group and 19 deaths out of 141 patients (13.4%) in the normal albumin–high CRP profile group. In the low albumin–normal CRP group, there were 46 deaths among 275 patients (20.0%), and in the low albumin–high CRP group, there were 32 deaths among 189 patients (16.9%). The albumin–CRP profiles did not attenuate the favourable effect of the upper tertiles of serum magnesium on the association with a reduction in HF, CHD, stroke, composite MACE and all-cause mortality (Table 2 and Supplementary Fig. S4).

### Subgroup analyses

**Figure 5:**
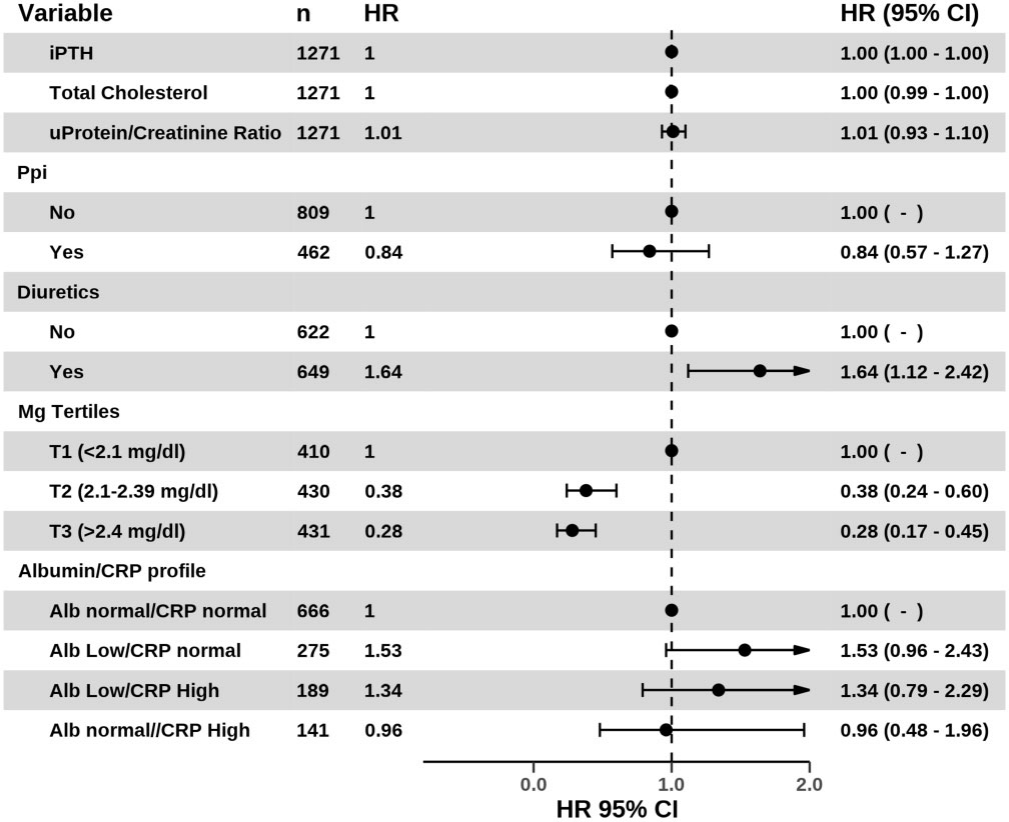
Forrest plot for the subgroup analysis of the association between baseline magnesium tertiles, nutritional/inflammatory profiles and MACE in patients with imaging results of echocardiography and evidence of LVH, LVEF, MAC and AVC. Only model 5 is displayed. CaA: calcium channel blockers; ADP: adenosine diphosphate receptor inhibitor.

Subgroup analyses were performed to explore potential modification of the risk association between serum magnesium and cardiovascular outcomes. This was done in a cohort of patients who underwent cardiac evaluation by echocardiography during follow-up, with adjustments made for the presence of LVH, LVEF, MAC and AVC. In brief, a total of 596 of 1271 subjects underwent echocardiographic assessment during the follow-up. LVH was detected in 285 of 596 (47.8%), AVC in 56 of 596 (9.3%) and MAC in 63 of 596 (10.5%). The mean LVEF was 65.1%. A total of 551 patients (92.4%) were classified as having heart failure with preserved ejection fraction, while 45 (7.5%) had heart failure with reduced ejection fraction. Cox regression models for subgroup analyses demonstrated that the positive effect of upper tertiles of magnesium and the association with a lower risk of fatal HF remained unchanged. However, an attenuation of the risk of CHD and stroke was observed in patients with magnesium levels in T2. In T3 patients, the association of serum magnesium with a reduced risk of CHD and stroke was maintained regardless of the presence of LVH, MAC or AVC (Supplementary Table S4). For the composite MACE and all-cause mortality, the association between upper tertiles of serum magnesium and a reduction in mortality was consistent with that for the primary outcome (Supplementary Table S4 and Fig. 5). As anticipated, preserved LVEF [HR 0.96 (95% CI 0.94–0.98)] was linked to a reduced likelihood of fatal HF. Conversely, MAC [HR 2.83 (95% CI 1.31–6.11)] and the use of calcium channel blockers [HR 3.35 (95% CI 1.89–5.92)] were associated with an elevated risk of death caused by HF. LVH was associated with a higher risk of fatal CHD [HR 2.71 (95% CI 1.09–6.66)]. None of these variables was associated with a higher risk of stroke in these subgroup analyses (Supplementary Table S4). The E-value supported the association between the upper tertiles of magnesium and risk reduction for the primary and secondary outcomes. For instance, for HF, the estimated E-value for T2 and T3 subjects in model 5 was 3.87 and 4.85, respectively, suggesting that residual confounding could negate the association of serum magnesium >2.1 mg/dl with a reduction in HF death if there was an unmeasured covariate with a relative risk ratio >3.87 and 4.85 for patients within each specific tertile (Supplementary Table S5).

## DISCUSSION

The results of the present study indicate that baseline serum magnesium concentration is associated with a significant reduction in mortality due to incident HF, CHD and stroke. The robust association between higher serum magnesium concentrations and a reduction in mortality from HF and CHD strongly suggests that the effect of upper-normal serum magnesium is independent of other traditional factors associated with mortality in CKD, including hypertension, diabetes, anaemia, hyperphosphataemia, hypoalbuminaemia, inflammation, MAC, AVC and LVH.

The majority of studies that have identified a correlation between magnesium deficiency, CVD and mortality have been conducted on dialysis patients [27–29]. The available information suggests that the association between serum magnesium levels and MACE is dependent on the presence of atherosclerosis and the development of vascular calcifications. Indeed, experimental studies in animals with vascular calcifications have demonstrated that dietary magnesium supplementation is an effective method for preventing and even partially reversing vascular calcifications [13]. The results of clinical studies have yielded contradictory results, thus perpetuating a debate regarding the existence of a significant relationship between magnesium and vascular damage. While one study failed to demonstrate a reduction in the progression of vascular calcifications following magnesium supplementation over 52 weeks [30], a separate study reported a reduction in the progression of coronary artery calcification (CAC) after the administration of magnesium oxide for 2 years. To the best of our knowledge, our study is the first to report that a magnesium concentration in the upper-normal range is associated with a lower incidence of death due to HF, CHD, stroke and composite MACE in patients with non-dialysis CKD stages 4 and 5.

Previous studies have demonstrated a correlation between low magnesium levels and the development of HF [31, 32]. Our results align with those of a prior study in diabetic patients that reported a decreased prevalence of HF in subjects with serum magnesium levels within the upper-normal range [12]. The precise role of magnesium deficiency in promoting ultrastructural changes in cardiac and/or vascular tissue and the onset of HF in patients with CKD is currently unknown.

It has been reported that in individuals with normal renal function, low serum magnesium levels are associated with CHD and carotid atherosclerosis [33, 34]. However, other studies have not identified such an association [35]. Our results demonstrate a significant reduction in the incidence of CHD, from 20.5 cases per 1000 person-years in patients with lower serum magnesium concentration (T1) to 5.6 cases per 1000 person-years in patients with the highest serum magnesium concentration (T3). These findings align with those of Sakaguchi *et al.* [14], suggesting that magnesium supplementation may reduce the progression of CAC and potentially the incidence of CHD.

With regard to the incidence of stroke, there was a notable reduction in incidence, with a decline from 14.6 to 1.4 per 1000 person-years (T1 versus T3). One study indicated that adjustments for other cardiovascular risk factors may negate the association between serum magnesium concentrations and the risk of stroke [36]. However, in our study, the inclusion of additional risk factors for CVD did not modify our results. Patients in the highest tertile of serum magnesium concentration had a reduced risk of stroke. Nevertheless, the beneficial effect of magnesium on the incidence of stroke was attenuated in patients in the second tertile after adjustment for tobacco use, beta-blockers and calcium channel blockers. The use of tobacco, the presence of diabetes and hypertension can all contribute to the development of microvascular and macrovascular disease, which may cause cardiovascular events [2]. Indeed, a correlation between low serum magnesium levels and the occurrence of microvascular events has been reported [12]. In our study, patients in the upper tertile of magnesium levels had significantly lower SBP, DBP and pulse pressure (PP). In a previous report, our group demonstrated that lower serum magnesium levels are associated with vascular dysfunction [37]. Of interest is our finding on the potential effect of magnesium supplementation in reducing pulse wave velocity [38], suggesting that magnesium is likely to improve vascular stiffness and BP [39]. Although the majority of cases of stroke can be attributed to atherosclerotic CVD, there is evidence to suggest that magnesium deficiency may contribute to cerebral vasospasm [40] and nuclear factor κB (NF-κB) activation, which increases the risk of stroke [41]. In rats with metabolic syndrome and CKD, a magnesium-enriched diet led to a reduction in NF-κB levels, which suggests that magnesium may have an antioxidant effect on the vasculature [37, 42]. It can therefore be inferred that serum magnesium may be associated with a reduction in MACE due to an improvement in SBP, DBP, PP and vascular stiffness. It remains unclear whether the beneficial effect of magnesium on cerebral vasculature is exclusive to cases where serum magnesium levels are within the upper-normal range. The evidence from the subgroup analysis of the effect of magnesium on the incidence of stroke is insufficient to draw robust conclusions because of the limited number of events.

In CKD, both inflammation and anorexia are common [43–45]. It has traditionally been assumed that magnesium deficiency is indicative of poor nutrition, chronic inflammation or both. This has led to serum magnesium levels being excluded as a direct cause of increased cardiovascular mortality [46–48]. Diagnosing PEW is straightforward in clinical practice. In accordance with the 2020 Kidney Disease Outcomes Quality Initiative (KDOQI) guidelines on nutrition in CKD, there is no compelling empirical evidence to substantiate the use of an exclusive tool for identifying or diagnosing PEW [43]. Consequently, the diagnosis of PEW is frequently based on biochemical parameters. In this regard, the majority of studies assessing the relevance of serum albumin as a marker of PEW, nutritional status and mortality have been conducted in dialysis-dependent CKD patients. There is a strong likelihood that inflammation affects nutritional status, although most of the evidence derives from cross-sectional studies in dialysis patients. In non-dialysis CKD patients, inflammation, as measured by serum CRP, is associated with suboptimal nutritional status. Novel methods to assess magnesium deficiency and PEW, such as the magnesium depletion score (MDS) [49] and the malnutrition-inflammation score (MIS) [50, 51], may be valuable approaches. However, the MDS, while useful for evaluating total body magnesium status, lacks insights into nutritional and inflammatory states and does not include recommendations for serum magnesium targets to mitigate MACE. A high MDS may be a surrogate for comorbidities beyond inflammation, malnutrition or CKD, and thus requires further validation in advanced CKD [52]. Similarly, the MIS, initially validated for dialysis patients [50] and also explored in non-dialysed CKD patients [53], is not currently recommended by the KDOQI guidelines for the evaluation of nutritional status in CKD patients other than those undergoing dialysis or transplant patients [43]. From a clinical perspective, the combination of albumin and CRP in our study enables clinicians to identify phenotypes of patients who are likely to exhibit disturbances in serum magnesium levels. Furthermore, our findings highlight the independent effect of serum magnesium in predicting reduced mortality beyond the influence of serum albumin and CRP levels.

Our study has some limitations. First, it was an observational study and therefore causality is limited due to residual confounding. Second, the time frame of evaluation is wide and some target values for certain variables included in the different analyses may have changed during the study period. We focused on baseline serum magnesium levels and did not consider longitudinal assessments, which could have revealed how changes in serum magnesium over time contribute to understanding its impact on mortality. In light of these considerations, we also acknowledge the possibility of time-varying confounding. Future validation studies, such as those analysing within-individual changes in serum magnesium over time, could contribute to a deeper understanding of the effects of magnesium on health and disease. The sequential analysis of >1000 patients, which encompassed a comprehensive consideration of potential confounders associated with CKD-related mortality and extensive subgroup analyses, served to reinforce the results. Furthermore, the calculation of E-values across all models adds support to our conclusions, indicating that substantial, unmeasured confounding factors would be required to negate the observed association between magnesium and reduced mortality. Yet, unmeasured confounding in the clinical setting can influence modifications of the results. Third, while acknowledging the inherent limitations of establishing causation in observational studies, our comprehensive analysis of established biomarkers, including serum albumin, BMI, total cholesterol and the combination of albumin and CRP as nutritional and inflammatory surrogates [43], partially compensates for unmeasured factors such as muscle mass, body fat percentage, fluid accumulation and serum pre-albumin levels, as well as the calculation of daily protein intake. The calculation of protein catabolic rate requires the collection of 24-hour urine samples, which are not routinely measured due to the inconvenience this poses for patients [22, 45]. Fourth, the majority of patients included were of European descent. Consequently, the generalisability of our findings may be limited. However, our results are consistent with those of other studies conducted in non-European populations [46]. Fifth, we employed a two-step verification process to confirm the outcomes and address the potential for information bias.

In conclusion, the findings of the present study consistently indicated that higher serum magnesium concentrations were associated with a substantial reduction in the incidence of fatal HF, CHD, stroke, composite MACE and all-cause mortality. Our findings highlight the necessity for a comprehensive assessment of magnesium homeostasis in non-dialysis CKD patients. It is noteworthy that the mortality risk reduction was observed in patients with serum magnesium concentrations within the upper-normal range, which may prompt a reconsideration of the recommended range for serum magnesium concentrations in CKD patients to prevent MACE. Further prospective clinical trials are necessary to ascertain the optimal serum magnesium concentrations for reducing MACE in patients with CKD.

In light of these findings, it is advisable for clinicians to consider low serum magnesium as an additional non-traditional risk factor for mortality, particularly given that the presence of other well-known risk factors for cardiovascular mortality does not substantially attenuate the benefits of an upper-normal serum magnesium concentration on the risk of mortality resulting from MACE.

## Supplementary Material

sfae390_Supplemental_File

## Data Availability

Data are available upon reasonable request.
