# Supplementary material for: Upper normal serum magnesium is associated with a reduction in incident death from fatal heart failure, coronary heart disease and stroke in non-dialysis patients with CKD stages 4 and 5
Source: Clin Kidney J. 2024 Dec 2;18(2):sfae390. doi: 10.1093/ckj/sfae390 (PMC11803307; doi:10.1093/ckj/sfae390)
Supplement: sfae390_Supplemental_File [file sfae390_supplemental_file.pdf]

SUPPLEMENTARY MATERIAL

Title: Upper normal serum magnesium is associated with a reduction in incident death from Fatal Heart Failure, Coronary Heart Disease, and Stroke in non-dialysis patients with CKD stages 4 and 5.

Authors: Cayetana Moyano-Peregrin, MD [a,b,c,d, ¶], Cristian Rodelo-Haad, MD; Ph.D. [a,b,c,d, ¶,*], Alejandro Martín-Malo, MD; Ph.D. [a,b,c,d], Juan Rafael Muñoz-Castañeda, Ph.D., [a,b,c,d], Raquel Ojeda, MD [a,b,c,d], Isabel Lopez-Lopez, MD [a,b,c,d], Mariano Rodríguez, MD; Ph.D., [a,b,c,d], Mª Victoria Pendon-Ruiz de Mier, MD; Ph.D [a,b,c,d], Rafael Santamaría, MD; Ph.D. [a,b,c,d, &] and Sagrario Soriano MD; Ph.D. [a,b,c,d, &]

- **Supplementary Methods.** Supporting analysis of Cox regression using serum magnesium as a continuous variable and subgroup analyses in patients with structural evaluation of the heart with echocardiography. ………………………………………….. **3**

- **Supplementary Table 1. Baseline serum Magnesium and risk of clinical outcomes. Models include serum magnesium as a numerical variable.** The results are reported as hazard ratios (95% confidence intervals) compared with the reference group……………………………………………………………………………….... **4**

- **Supplementary Table 2. Characteristics of the population included according to secondary outcomes. ………….………….. 5**

- **Supplementary Table 3. Patient characteristics according to their nutritional and inflammatory clinical profiles………………………..…………………………….... 6**

- **Supplementary Table 4. Subgroup analyses of patients with echocardiography.. 8**

- **Supplementary Table 5. Sensitivity Analysis for Unmeasured confounding. E-value** assessed the minimum strength of potential unmeasured confounding required to negate the effect of Mg on mortality……...…………………………………………….… **9**

- **Supplementary Figure 1. Flowchart of the study patients…………………….….10**

- **Supplementary Figure 2. Correlograms show the correlations between the different variables analyzed. ……………………………………………..…..…...… 11**

- **Supplementary Figure 3. Baseline serum magnesium (Mg) according to different albumin/CRP clinical profiles.** Least square (L.S.) means of Mg by albumin/CRP profiles adjusted for eGFR, age, and serum phosphate………..……..…………….…... **12**

- **Supplementary Figure 4. Forrest plot for the risk association of baseline magnesium tertiles, and nutritional/inflammatory profiles with MACE…….……...13**

Supplementary Methods.

Supporting analysis of Cox regression using serum Mg as a continuous variable.

Cox regression models were also constructed by including serum magnesium level as a continuous variable. These models included interaction terms to assess the effect modification. The final models were as follows: **Model 1** adjusted for Sex, Age, diabetes, hypertension, BMI, and tobacco use. M**odel** 2 adjusted for significant variables in model 1 (mg tertiles and BMI), sPhosphate, haemoglobin + the use of oral iron preparations, calcium binders, RAAS acting agents, statins, aspirin, and beta-blockers**. Model 3** adjusted for significant variables in model 2 (mg tertiles, BMI), iPTH, sCalcium, hs-CRP, magnesium*hs-CRP interaction, diuretics, VKA and calcium channel blockers. **Model 4** adjusted for significant variables in model 3 (mg tertiles, iPTH, BMI and diuretics), eGFR, serum albumin, hs-CRP, albumin*hs-CRP interaction, magnesium*albumin*hs-CRP interaction, total cholesterol, the use of Ppi. **Model 5** adjusted for significant variables in model 4 (mg tertiles, iPTH, total cholesterol and diuretics), uProtein-to-Creatinine Ratio, the use of PPIs, PPIs*diuretics interaction and nutritional/inflammatory profiles (normal albumin / normal CRP as the reference category).

Subgroup analyses, including patients with echocardiographic evidence of LVH, LVEF, MAC and AVC, included the following models: **Model 1** adjusted for mg tertiles, LVH, LVEF, tobacco use, and calcium channel blockers use**.** M**odel** 2 adjusted for significant variables in model 1 (mg tertiles, LVH, LVEF and the use of calcium channel blockers), the use of VKA, aspirin and the presence of MAC. **Model 3** adjusted for significant variables in model 2 (mg tertiles, LVH, LVEF, the use of calcium channel blockers and the presence of MAC), the use of beta-blockers. **Model 4** adjusted for significant variables in Model 3 (mg tertiles, LVH, LVEF, the use of calcium channel blockers and the presence of MAC), the presence of aortic valve calcifications (AVC); **Model 5** adjusted for significant variables in model 2 (mg tertiles, LVH, LVEF, the use of calcium channel blockers and the presence of MAC), the use of ADP and Nutritional/inflammatory profiles (normal albumin / normal CRP as the reference category).

Supplementary Table 1. Baseline serum magnesium and risk of clinical outcomes. The results are reported as hazard ratios (95% confidence intervals).

**Cause of Death**	**Unadjusted**	**Model 1**	**Model2**	**Model 3**	**Model 4**	**Model 5 Nutritional/Inflammatory profiles**
**Heart Failure (66 events)**						
sMagnesium (mg/dl)	0.59 (0.45—0.77) ***	0.64 (0.50—0.83) ***	0.65 (0.50—0.85) ***	0.64 (0.48—0.86) **	0.67 (0.49—0.92) *	0.62 (0.47—0.83) **
**Coronary Heart Disease (38 events)**						
sMagnesium (mg/dl)	0.49 (0.35—0.70) ***	0.56 (0.40—0.80) **	0.56 (0.40—0.80) **	0.51 (0.34—0.75) **	0.48 (0.32—0.74) **	0.47 (0.32—0.70) ***
**Stroke (23 events)**						
sMagnesium (mg/dl)	0.57 (0.35—92) *	0.63 (0.39—1.02)	0.65 (0.39—1.09)	0.44 (0.25—0.81) *	0.50 (0.25—1.02)	0.41 (0.23—0.74) **
**MACE (127 events)**						
sMagnesium (mg/dl)	0.55 (0.46—0.67) ***	0.61 (0.51—0.74) ***	0.63 (0.52—0.76) ***	0.57 (0.47—0.70) ***	0.58 (0.47—0.73) ***	0.54 (0.44—0.67) ***
**All-cause death (186 events)**						
sMagnesium (mg/dl)	0.60 (0.51—0.70) ***	0.65 (0.56—0.76) ***	0.67 (0.57—0.78) ***	0.64 (0.54—0.76) ***	0.63 (0.52—0.75) ***	0.60 (0.51—0.71) ***

Model 1 adjusted for Sex, Age, diabetes, hypertension, BMI, and tobacco use; M**odel** 2 adjusted for significant variables in model 1 (mg tertiles and BMI) + sPhosphate + hemoglobin + the use of oral iron preparations, calcium binders, RAAS acting agents, statins, aspirin and beta-blockers**; Model 3** adjusted for significant variables in model 2 (mg tertiles, BMI) + iPTH + sCalcium + hs-CRP + + mg*hs-CRP interaction + diuretics + VKA and calcium channel blockers; **Model 4** adjusted for significant variables in model 3 (mg tertiles, iPTH, BMI and diuretics) + eGFR + serum albumin + hs-CRP + albumin*hs-CRP interaction + mg*albumin*hs-CRP interaction + total cholesterol + the use of Ppi; **Model 5** adjusted for significant variables in model 4 (mg tertiles, iPTH, total cholesterol and diuretics) + uProtein-to-Creatinine Ratio + the use of Ppi + PPi*diuretics interaction and Nutritional/inflammatory profiles (Alb normal / CRP normal as reference). * p<0.05, ** p<0.01 and *** p<0.001; [a] Data in parentheses displayed as 95% IC.

Supplementary Table 2. Characteristics of the population included.

	All n=1271	Died n=186	Survived n= 1085	P	smd
**Sex (n, %)**					
Female	488 (38.4)	73 (39.2)	415 (38.2)	0.807	0.020
Male	783 (61.6)	113 (60.8)	670 (61.8)		
**DM (n, %)**					
No	836 (65.8)	117 (62.9)	719 (66.3)	0.403	0.070
Yes	435 (34.2)	69 (37.1)	366 (33.7)		
**HTN (n, %)**					
No	413 (32.5)	53 (28.5)	360 (33.2)	0.236	0.102
Yes	858 (67.5)	133 (71.5)	725 (66.8)		
**Tobacco (n, %)**					
No	558 (65.3)	109 (59.9)	449 (64.4)	0.297	0.093
Yes	321 (36.5)	73 (40.1)	248 (35.6)		
**Follow-Up (Years)***	2.14 (0.79—4.33)	1.46 (0.50—2.80)	2.24 (0.86—4.65)	<0.001	0.515
**Age (Years)** #	72.10 (13.31)	74.54 (10.07)	71.69 (13.75)	0.007	0.237
**eGFR by CKD-EPI (mil/min/1.73m^2)** #	15.03 (4.78)	16.39 (5.06)	14.86 (4.75)	<0.001	0.312
**BMI (kg/m)** #	29.15 (4.48)	29.45 (3.75)	29.10 (4.59)	0.320	0.084
**SBP (mmHg)** #	147.15 (22.04)	146.51 (21.07)	147.26 (22.21)	0.672	0.034
**DBP (mmHg)** #	79.65 (13.00)	77.26 (10.73)	80.06 (13.31)	0.007	0.231
**MBP (mmHg)** #	102.15 (13.70)	100.35 (12.59)	102.46 (13.86)	0.052	0.159
**PP (mmHg)** #	67.70 (19.45)	69.25 (17.25)	67.44 (19.80)	0.240	0.098
**Hb (g/l)** #	11.54 (1.59)	11.30 (1.47)	11.58 (1.61)	0.022	0.188
**Hct (%)** #	34.79 (4.64)	34.27 (4.26)	34.88 (4.70)	0.097	0.136
**calcium (mg/dl)** #	9.19 (0.72)	9.17 (0.67)	9.19 (0.73)	0.726	0.029
**sPhosphate(mg/dl)** #	4.43 (0.80)	4.33 (0.82)	4.44 (0.80)	0.078	0.139
**sMagnesium (mg/dl)** #	2.21 (0.42)	2.06 (0.46)	2.24 (0.41)	<0.001	0.419
**iPTH (pg/ml)** *	150.0 (90.20—241.0)	139.90 (89.6—230.3)	151.5 (90.4—241.7)	0.677	0.087
**Total cholesterol (mg/dl)** #	176.42 (41.99)	169.43 (38.8)	177.62 (42.4)	0.020	0.114
**hs-CRP (mg/l)** *	4.40 (1.80—10.25)	4.55 (2.40, 11.7)	4.30 (1.70, 10.2)	0.070	0.103
**Albumin (g/l)** #	3.90 (0.51)	3.85 (0.47)	3.91 (1.07)	0.124	0.126
**uProtein/Creatinine ratio (mg/g)** #	1.76 (2.61)	1.54 (2.30)	1.79 (2.65)	0.215	0.103
**RAASi (n, %)**					
No	543 (42.7)	76 (40.9)	468 (43.1)	0.525	0.057
Yes	728 (57.3)	111 (59.7)	617 (56.9)		
**Diuretics (n, %)**					
No	622 (48.9	76 (40.9)	546 (50.3)	0.021	0.191
Yes	649 (51.1)	110 (59.1)	539 (49.6)		
**Ca-Based_Binder (n, %)**					
No	1117 (87.9)	168 (90.3)	949 (64.3)	0.326	0.091
Yes	154 (12.1)	18 (9.7)	136 (12.5)		
**PPi (n, %)**					
No	809 (63.7)	111 (59.6)	698 (64.3)	0.256	0.096
Yes	462 (36.3)	75 (40.3)	387 (35.7)		
**Iron Supplements (n, %)**					
No	904 (71.1)	116 (62.4)	788 (72.6)	0.006	0.220
Yes	367 (28.9)	70 (37.6)	297 (27.4)		
**Statins (n, %)**				0.165	0.123
No	406 (40.0)	62 (35.0)	344 (41.0)		
Yes	610 (60.0)	115 (65.0)	495 (59.0		
**Aspirin (n, %)**				0.042	0.174
No	609 (69.1)	114 (62.6)	495 (59.0)		
Yes	272 (30.9)	68 (37.4)	204 (29.2)		
**ADP antagonist (n, %)**				0.488	0.068
No	892	161 (88.5)	631 (90.5)		
Yes	87	21 (11.5)	66 (9.5)		
**VKA (n, %)**				0.323	0.091
No	809	164 (90.1)	645 (92.7)		
Yes	69	18 (9.9)	51 (7.3)		

**Calcium-channel blockers (n, %)**				<0.001	0.402
No	700	120 (65.9)	580 (83.1)		
Yes	180	62 (34.1)	118 (16.9)		
**Beta Blockers (n, %)**				0.422	0.074
No	609	121 (66.5)	488 (69.9)		
Yes	271	61 (33.5)	210 (30.1)		

Abbreviations: D.M., diabetes mellitus; HTN, hypertension; eGFR, estimated glomerular filtration rate by the CKD-EPI formula; BMI, body mass index (calculated as weight in kilograms divided by height in meters squared); SBP, systolic blood pressure; DBP, diastolic blood pressure; MBP, mean arterial blood pressure (calculated as DBP + 1/3 [SBP – DBP); P.P., pulse pressure (calculated as the difference between the SBP and DBP); Hb, hemoglobin; Hct, hematocrit; iPTH, intact parathyroid hormone; hs-CRP, high sensitive C-reactive protein; RAASi, renin-angiotensin-aldosterone system inhibitors; Ca-based binders, calcium-based phosphate binders; PPi, proton pump inhibitors; ; ADP antagonist, Adenosine diphosphate receptor inhibitor; VKA, vitamin K antagonist

* IQR, Median and interquartile range; [#] Mean and S.D., standard deviation; smd, standardized mean difference

Supplementary Table 3. Characteristics of patients according to nutritional and inflammatory clinical profiles

	Nutritional / Inflammatory Clinical Profiles					
	Albumin normal/CRP Low n=666	Albumin Low/CRP High n=189	Albumin Low/CRP Low n=275	Albumin normal/CRP High n=141	P	smd
**Gender (n, %)**						
**Female**	252 (37.8)	77 (40.7)	116 (42.2)	43 (30.5)	0.114	0.132
**Male**	414 (62.2)	112 (59.3)	159 (57.8)	98 (69.5)		
**D.M. (n, %)**						
**No**	465 (69.8)	119 (63.0)	156 (56.7)	96 (68.1)	0.001	0.155
**Yes**	201 (30.2)	70 (37.0)	119 (43.3)	45 (31.9)		
**HTN (n, %)**						
**No**	247 (37.1)	52 (27.5)	74 (26.9)	40 (28.4)	0.004	0.113
**Yes**	419 (62.9)	137 (72.5)	201 (73.1)	101 (71.6)		
**Tobacco (n, %)**						
**No**	274 (64.0)	102 (60.7)	118 (66.7)	64 (60.4)	0.610	0.077
**Yes**	154 (36.0)	66 (39.3)	59 (33.3)	42 (39.6)		
**Follow-Up (Years) (median [IQR])**	2.49 [1.21, 4.73]	1.57 [0.38, 3.75]	1.43 [0.51, 3.32]	1.66 [0.66, 3.43]	<0.001	0.157
**Age (mean, SD)**	71.45 (13.67)	75.08 (12.57)	71.63 (13.44)	72.13 (11.79)	0.009	0.149
**eGFR by CKD-EPI (mil/min/1.73m^2) (mean, SD)**	15.41 (4.82)	14.35 (5.02)	14.70 (4.51)	14.77 (4.67)	0.021	0.113
**BMI (kg/m, mean, SD)**	29.06 (4.46)	29.06 (4.52)	29.32 (4.46)	29.35 (4.54)	0.789	0.043
**SBP (mmHg, mean, SD)**	145.82 (21.80)	147.28 (21.71)	151.66 (23.49)	144.46 (19.57)	0.001	0.175
**DBP (mmHg, mean, SD)**	79.81 (13.19)	78.51 (12.30)	81.28 (12.73)	77.21 (13.14)	0.012	0.175
**MBP (mmHg, mean, SD)**	101.81 (13.83)	101.43 (13.05)	104.74 (13.82)	99.63 (13.00)	0.001	0.195
**PP (mmHg, mean, SD)**	66.20 (19.00)	68.77 (19.85)	70.84 (20.52)	67.26 (18.21)	0.008	0.132
**Hb (g/l, mean, SD)**	11.91 (1.56)	10.67 (1.42)	11.35 (1.53)	11.33 (1.53)	<0.001	0.414
**Hct (%, mean, SD)**	35.88 (4.54)	32.35 (4.34)	34.13 (4.38)	34.23 (4.45)	<0.001	0.401
**sCalcium (mg/dl, mean, SD)**	9.39 (0.64)	8.85 (0.78)	8.89 (0.69)	9.26 (0.70)	<0.001	0.474
**sPhosphate (mg/dl, mean, SD)**	4.35 (0.78)	4.50 (0.82)	4.58 (0.79)	4.40 (0.87)	<0.001	0.165

**sMagnesium (mg/dl, mean, SD)**	2.24 (0.39)	2.14 (0.47)	2.19 (0.45)	2.24 (0.40)	0.010	0.142
**iPTH (pg/ml, median [IQR])**	148.0 (88.8— 240.6]	150.0 (97.0—246.9)	157.5 (85.5—243.9)	152.5 (103.2—229.1)	0.827	0.071
**Total cholesterol (mg/dl, mean, SD)**	176.91 (39.57)	168.83 (43.35)	180.35 (46.00)	176.61 (42.24)	0.034	0.134
**hs-CRP (mg/l, median [IQR])**	2.50 [1.20, 4.80]	22.00 [15.30, 31.00]	3.70 [1.55, 5.45]	17.50 [13.00, 26.40]	<0.001	1.817
**sAlbumin (g/l, mean, SD)**	4.19 (0.32)	3.36 (0.39)	3.41 (0.33)	4.19 (0.28)	<0.001	1.653
**uProtein/Creatinine ratio (mg/g, mean, SD)**	1.10 (1.64)	2.56 (3.35)	3.07 (3.57)	1.19 (1.45)	<0.001	0.448
**RAASi (n, %)**						
**No**	278 (41.7)	91 (48.1)	113 (41.1)	61 (43.3)	0.415	0.076
**Yes**	388 (58.3)	98 (51.9)	162 (58.9)	80 (56.7)		
**Furosemide (n, %)**						
**No**	339 (50.9)	88 (46.6)	127 (46.2)	68 (48.2)	0.509	0.053
**Yes**	327 (49.1)	101 (53.4)	148 (53.8)	73 (51.8)		
**Ca-Based_Binder (n, %)**						
**No**	598 (89.8)	159 (84.1)	233 (84.7)	127 (90.1)	0.046	0.114
**Yes**	68 (10.2)	30 (15.9)	42 (15.3)	14 (9.9)		
**PPi (n, %)**						
**No**	442 (66.4)	109 (57.7)	181 (65.8)	77 (54.6)	0.014	0.149
**Yes**	224 (33.6)	80 (42.3)	94 (34.2)	64 (45.4)		
**Iron Supplements (n, %)**						
**No**	476 (71.5)	141 (74.6)	193 (70.2)	94 (66.7)	0.451	0.092
**Yes**	190 (28.5)	48 (25.4)	82 (29.8)	47 (33.3)		

Abbreviations: D.M., diabetes mellitus; HTN, hypertension; eGFR, estimated glomerular filtration rate by the CKD-EPI formula; BMI, body mass index (calculated as weight in kilograms divided by height in meters squared); SBP, systolic blood pressure; DBP, diastolic blood pressure; MBP, mean arterial blood pressure (calculated as DBP + 1/3 [SBP – DBP); P.P., pulse pressure (calculated as the difference between the SBP and DBP); Hb, hemoglobin; Hct, hematocrit; iPTH, intact parathyroid hormone; hs-CRP, high sensitive C-reactive protein; RAASi, renin-angiotensin-aldosterone system inhibitors; Ca-based binders, calcium-based phosphate binders; PPi, proton pump inhibitors. IQR, interquartile range; S.D., standard deviation; smd, standardized mean difference.

Supplementary Table 4. Subgroup analyses of patients with echocardiography.

Cause of Death	Model 1	Model 2	Model 3	Model 4	Model Nutritional/Inflammatory profiles
**Heart Failure (58 events)**					
T1	Reference	Reference	Reference	Reference	Reference
T2	0.53 (0.28—0.98) *	0.53 (0.28—0.99) *	0.53 (0.28—0.99) *	0.52 (0.28—0.96) *	0.52 (0.28—0.98) *
T3	0.33 (0.16—0.68) **	0.31 (0.15—0.64) **	0.31 (0.15—0.64)**	0.31 (0.15—0.64) **	0.33 (0.16—0.69) **
**Coronary Heart Disease (30 events)**					
T1	Reference	Reference	Reference	Reference	Reference
T2	0.43 (0.16—1.17)	0.40 (0.15—1.11)	0.42 (0.15—1.16)	0.43 (0.15—1.17)	0.40 (0.14—1.11)
T3	0.29 (0.11—0.78)*	0.29 (0.10—0.79) *	0.29 (0.11—0.79) *	0.29 (0.10—0.78) *	0.28 (0.10—0.79) *
**Stroke (15 events)**					
T1	Reference	Reference	Reference	Reference	Reference
T2	0.27 (0.05—1.29)	0.26 (0.05—1.42)	0.27 (0.05—1.35)	0.25 (0.05—1.30)	0.28 (0.04—1.92)
T3	0.16 (0.02—1.0) *	0.16 (0.02—1.13)	0.15 (0.02—1.0)*	0.15 (0.02—1.0) *	0.18 (0.01—1.70)
**MACE (103 events)**					
T1	Reference	Reference	Reference	Reference	Reference
T2	0.45 (0.28—0.73) ***	0.45 (0.28—0.73) ***	0.46 (0.28—0.74) ***	0.42 (0.26—0.69) ***	0.46 (0.29—0.75) ***
T3	0.29 (0.17—0.49) ***	0.28 (0.16—0.47) ***	0.28 (0.16—0.48) ***	0.28 (0.16—0.47) ***	0.29 (0.17—0.50) ***
**All-cause Mortality (135 events)**					
T1	Reference	Reference	Reference	Reference	Reference
T2	0.44 (0.29—0.68) ***	0.44 (0.29—0.68) ***	0.45 (0.29—0.68) ***	0.42 (0.27—0.65) ***	0.45 (0.29—0.69) ***
T3	0.23 (0.23—0.55) ***	0.35 (0.23—0.55) ***	0.35 (0.23—0.55) ***	0.35 (0.23—0.55) ***	0.36 (0.23—0.57) ***

Model 1 adjusted for **mg tertiles + LVH +LVEF + tobacco use and calcium channel blockers use;** M**odel** 2 adjusted for significant variables in model 1 (mg tertiles, LVH, LVEF and the use of calcium channel blockers) + the use of VKA, aspirin and the presence of MAC; **Model 3** adjusted for significant variables in model 2 (mg tertiles, LVH, LVEF, the use of calcium channel blockers and the presence of MAC) + the use of beta-blockers; **Model 4** adjusted for significant variables in model 3 (mg tertiles, LVH, LVEF, the use of calcium channel blockers and the presence of MAC) + the presence of aortic valve calcifications (AVC); **Model 5** adjusted for significant variables in model 2 (mg tertiles, LVH, LVEF, the use of calcium channel blockers and the presence of MAC) + the use of ADP and Nutritional/inflammatory profiles (Alb normal / CRP normal as reference). ***** p<0.05, ****** p<0.01 and ******* p<0.001; [a] Data in parentheses displayed as 95% IC.

Supplementary Table 5. Sensitivity Analysis for Unmeasured confounding. E-value calculation evaluates the strength that an unmeasured or unknown covariate should have to negate the association between the covariate of interest and the outcome. For the current study, we show the e-value for the main models 4 and model 5 (Table 2) given that both models include the strongest confounders associated with mortality in CKD patients. T1= <2.1 mg/dl, T2=2.1—2.39 mg/dl, T3= >2.4 mg/dl.

Cause of Death	Model 4	Model 5 Nutritional/Inflammatory profiles
	**E-value (CI)***	**E-value (CI)***
**HF**		
T2	3.33 (1.,0)	3.87 (1.6)
T3	4.7 (1.81)	4.85 (2.12)
**CHD**		
T2	5.91 (2.04)	5.91 (2.04)
T3	6.6 (2.26)	6.35 (2.26)
**Stroke**		
T2	5.51 (1.0)	7.15 (1.39)
T3	32.8 (1.56)	39.4 (2.84)
**MACE**		
T2	4.31 (2.45)	4.7 (2.72)
T3	6.6 (3.59)	6.6 (3.87)
**All-Cause**		
T2	3.77 (2.35)	4.08 (2.55)
T3	4.57 (2.84)	4.85 (3.11)

FIGURES

Supplementary Figure 1.

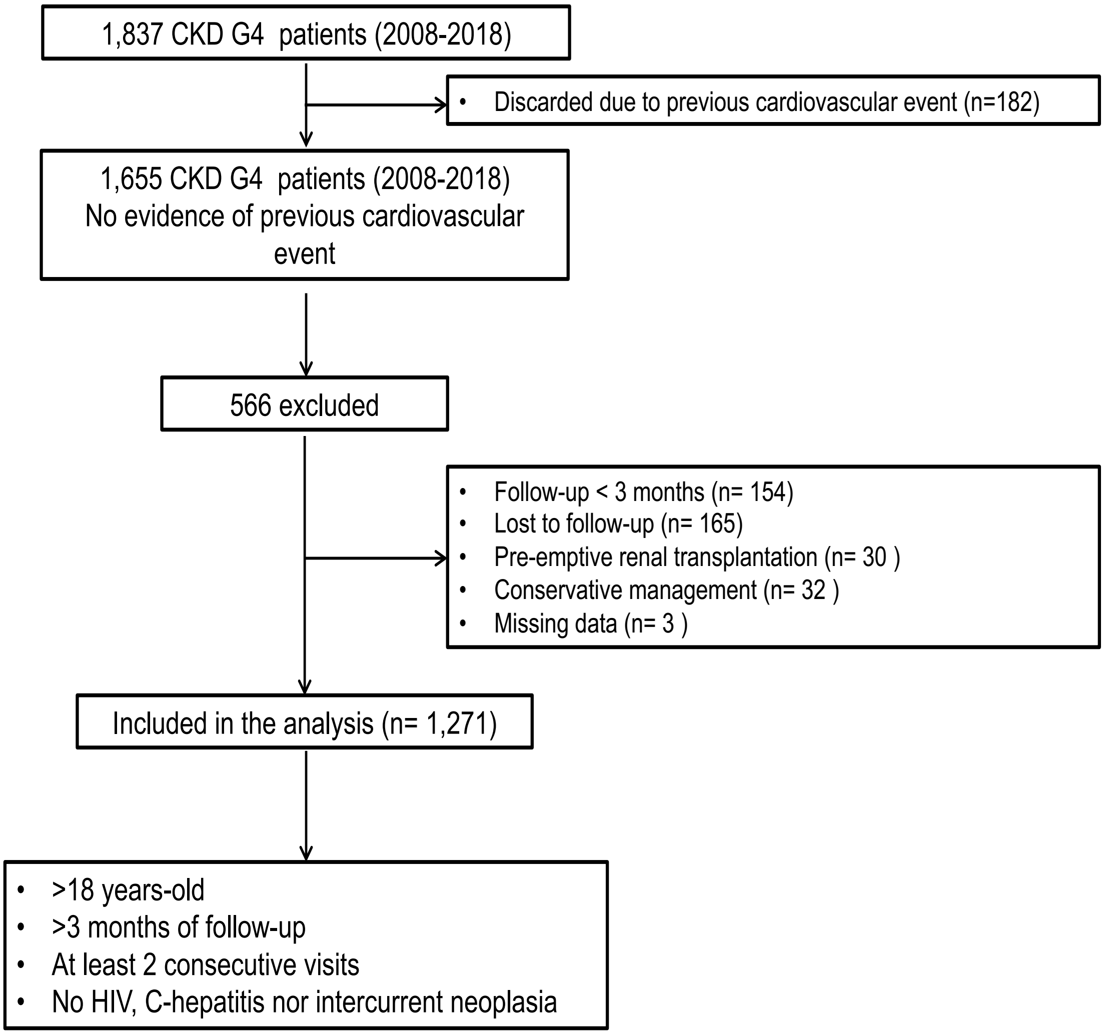

Supplementary Figure 2.

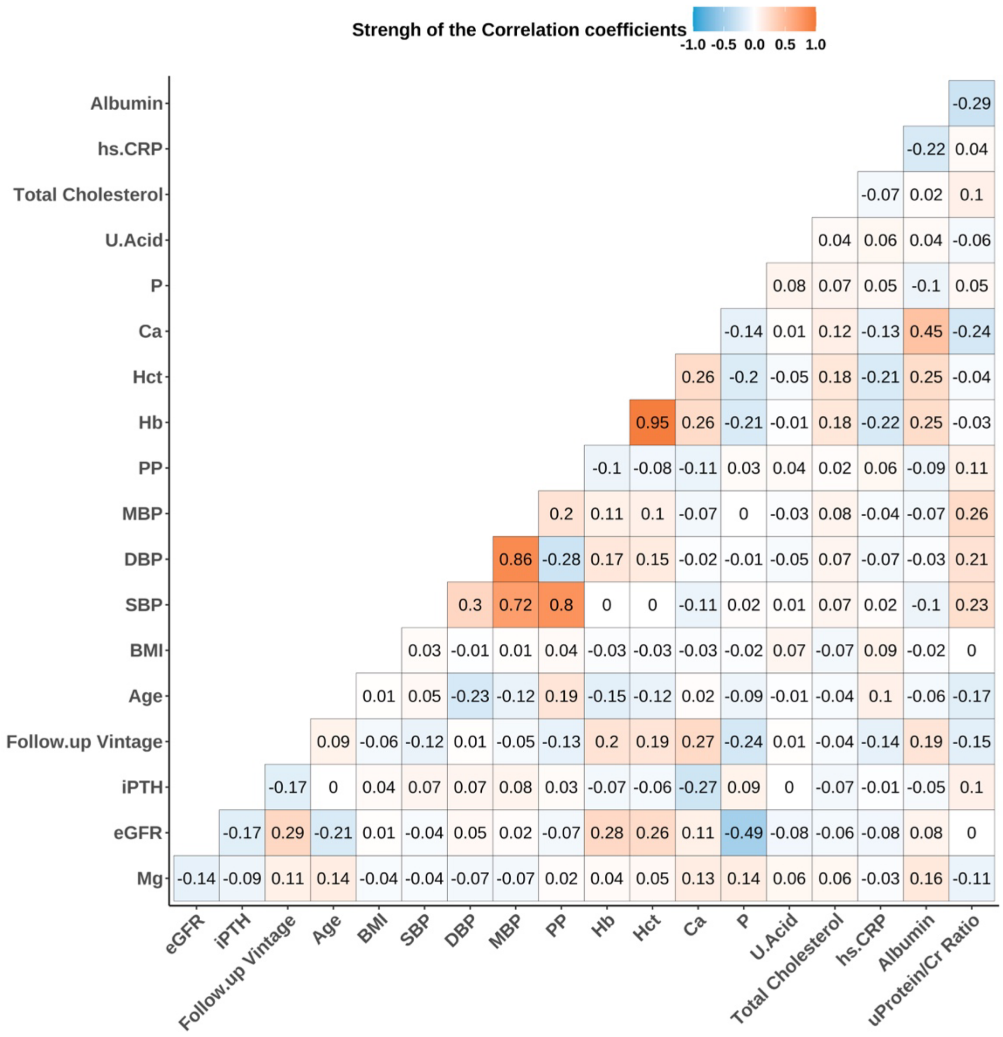

Supplementary Figure 2. Correlogram showing the correlations between the different variables analysed. The color intensity represents the strength of the Spearman's correlation coefficient. Abbreviations: Mg, serum magnesium; eGFR, estimated glomerular filtration rate by the CKD-EPI formula; iPTH, intact parathyroid hormone; BMI, body mass index (calculated as weight in kilograms divided by height in meters squared); SBP, systolic blood pressure; DBP, diastolic blood pressure; MBP, mean arterial blood pressure (calculated as DBP + 1/3 [SBP – DBP); P.P., pulse pressure (calculated as the difference between the SBP and DBP); Hb, hemoglobin; Hct, hematocrit; Ca, serum calcium; P, serum phosphate; U.acid, uric acid hs-CRP, high-sensitivity C-reactive protein).

Supplementary Figure 3.

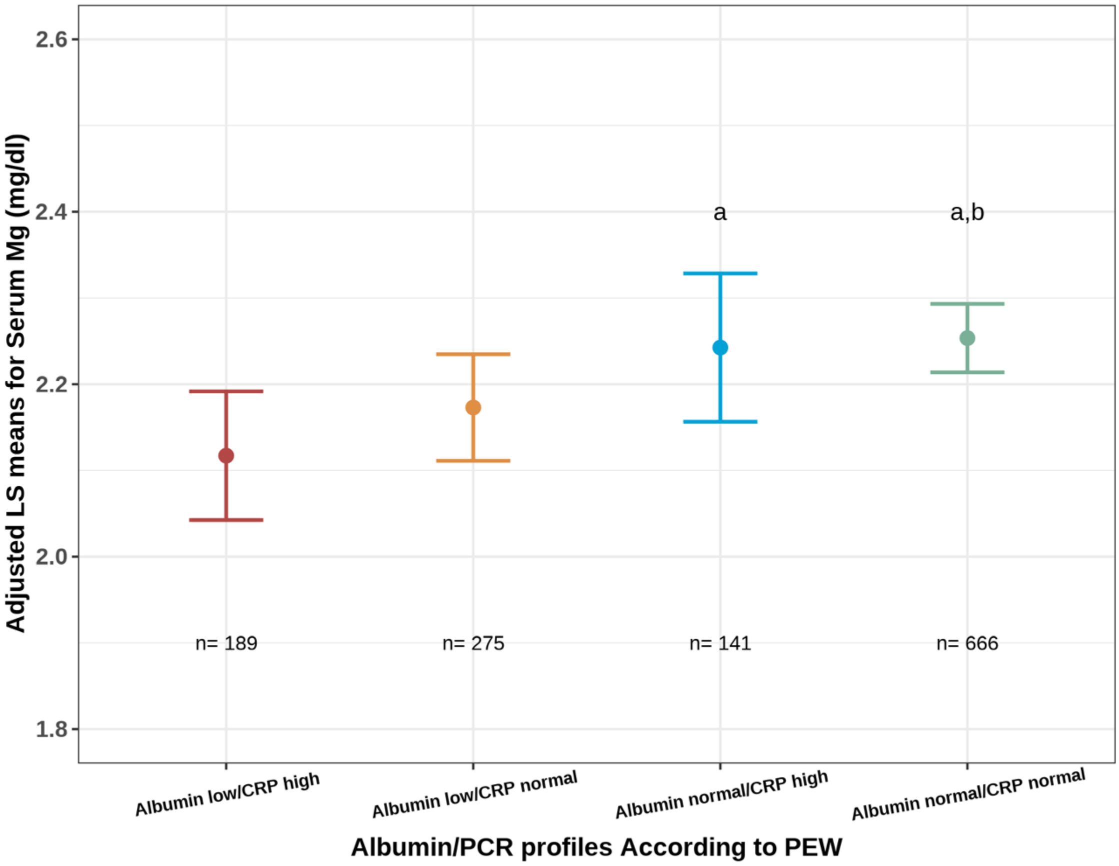

Supplementary Figure 4. Baseline serum magnesium according to different albumin/CRP clinical profiles. Least square (L.S.) means of Mg by albumin/CRP profiles adjusted for eGFR by CKD-EPI, serum phosphate, and age. Dot points represent mean values, and whiskers represent 95% confidence intervals. [a] $p<0.05$ compared to Albumin Low/CRP High, [b] $p<0.05$ compared to Albumin Low/CRP normal.

Supplementary Figure 4.

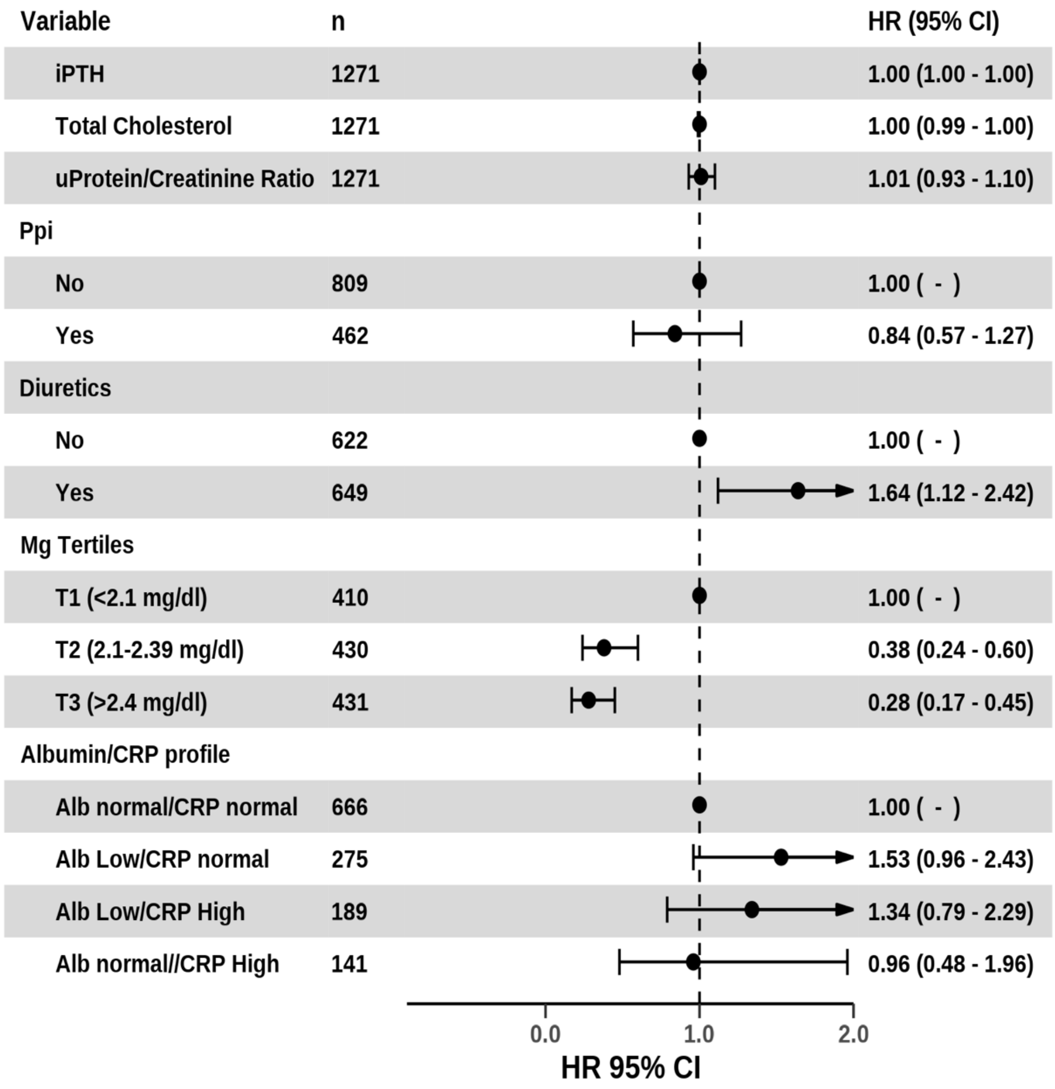

Supplementary Figure 4. Hazard ratios for the risk association of magnesium tertiles, and nutritional/inflammatory profiles with MACE. Fully adjusted model (Model 5). Abbreviations: Ppi, Proton pump inhibitors; iPTH, intact parathyroid hormone; uP/Cr ratio, Urinary protein-to-creatinine ratio. HR, Hazard Ratio with 95% confidence interval.
